# A 2-year prospective multicenter study of ultrasound cyclo plasty for glaucoma

**DOI:** 10.1038/s41598-021-92233-9

**Published:** 2021-06-16

**Authors:** Giuseppe Giannaccare, Marco Pellegrini, Federico Bernabei, Lara Urbini, Fulvio Bergamini, Lorenzo Ferro Desideri, Alessandro Bagnis, Francesco Biagini, Paola Cassottana, Chiara Del Noce, Adriano Carnevali, Vincenzo Scorcia, Carlo E. Traverso, Aldo Vagge

**Affiliations:** 1 Department of Ophthalmology, University Magna Graecia of Catanzaro, Viale Europa, 88100 Germaneto, Catanzaro Italy; 2 Department of Ophthalmology, Ospedali Privati Forlì “Villa Igea”, Forlì, Italy; 3 Istituto Internazionale per la Ricerca e Formazione in Oftalmologia (IRFO), Forlì, Italy; 4 Service d’ophtalmologie, Ophtalmopôle de Paris, Hospital Cochin, Paris, France; 5 Department of Ophthalmology, Scientific Institute Capitanio Hospital-IRCCS Foundation-Istituto Auxologico Italiano, Milan, Italy; 6 Eye Clinic, DiNOGMI, University of Genoa, Genoa, Italy

**Keywords:** Outcomes research, Ocular hypertension

## Abstract

Ultrasound cyclo plasty (UCP) is a recently developed surgical technique for glaucoma allowing a selective and controlled coagulation of the ciliary body. We herein investigated the long-term efficacy and safety of UCP for the treatment of glaucoma. This prospective study included patients with primary and secondary glaucoma. All surgeries were performed using the EyeOP1 device (Eye Tech Care, Rillieux-la-Pape, France). Sixty-six patients were included, and 60 completed regularly the 2-year follow-up. Preoperative IOP was 28.5 ± 9.6 mmHg and significantly decreased to 17.0 ± 5.4 at 2 years (*p* < 0.001). The daily number of both hypotensive eye drops and acetazolamide tablets decreased significantly (respectively, from 2.6 ± 1.1 to 1.7 ± 1.2 and from 0.7 ± 0.8 to 0.2 ± 0.5; both *p* < 0.001). At 2 years, 68.1% of patients met the definition of qualified success (IOP < 21 mmHg regardless of glaucoma medications) and 10.3% of patients met the definition of complete success (IOP < 21 mmHg without glaucoma medications). No major intra- or postoperative complications occurred; however, 15 eyes required additional glaucoma surgery. These results suggest that UCP is an effective and safe procedure to reduce IOP in glaucoma patients through a 2-year follow-up period.

## Introduction

Glaucoma represents one of the leading causes of irreversible blindness worldwide^1^. As a chronic and progressive condition, it may significantly affect quality of life^2–4^. Lowering the intraocular pressure (IOP) is considered the main strategy for the treatment of glaucoma, albeit neuroprotection has recently emerged as an additional therapeutic approach to prevent or slow the progression of optic nerve damage^5^. Surgery is usually indicated when the disease remains uncontrolled despite non-invasive therapy that includes either hypotensive topical medications or laser procedures. Although various surgical techniques with different amount of invasiveness have been proposed, trabeculectomy still represents the gold standard of treatment^6^. Traditionally, ciliary body destruction has been considered a last-resort option to control IOP in refractory or advanced glaucoma cases with poor visual potential due to the high rate of complications^7–9^.

Ultrasound cyclo plasty (UCP) is a relatively recently developed technique able to achieve a selective and controlled coagulation of the ciliary body by means of high-intensity focused ultrasound while sparing the adjacent ocular structures^10^. Various initial reports of UCP have demonstrated positive outcomes for reducing IOP in patients with different types of glaucoma^11–22^. However, to date the long-term outcomes of safety and efficacy of the procedure remain largely undetermined. The purpose of this study was to report the 2-year outcomes of UCP procedures performed in 3 Italian glaucoma centers.

## Results

A total of 66 eyes of 66 patients were enrolled in the study. Demographic and baseline clinical characteristics are reported in Table 1. Eight patients (12.1% of the total) had previously undergone selective laser trabeculoplasty, while previous ocular surgery included cataract phacoemulsification in 45 patients (68.2%), trabeculectomy in 13 (19.7%), pars plana vitrectomy in 7 (10.6%), laser cyclophotocoagulation in 3 (4.5%), EX-PRESS implant in 2 (3.9%), deep sclerectomy in 1 (1.5%), XEN gel stent implant in 1 (1.5%) and penetrating keratoplasty in 1 (1.5%). In total, 7 patients (10.6%) had a preoperative IOP < 21 mmHg but required surgery because they were intolerant to glaucoma medications.

**Table 1:** Demographical and clinical characteristics of patients treated with ultrasound cyclo plasty.

Characteristic	Patients (n = 66)
Age (years)	72.4 ± 13.6
Gender (male/female)	28/38
**Type of glaucoma (%)**	
Primary open-angle angle glaucoma	36 (54.5%)
Primary angle-closure glaucoma	11 (16.6%)
Exfoliative glaucoma	9 (13.6%)
Neovascular glaucoma	7 (10.6%)
Congenital glaucoma	1 (1.5%)
Pigmentary glaucoma	1 (1.5%)
Post-surgical glaucoma	1 (1.5%)
Visual field (dB)	− 18.4 ± 8.8
Lens status (pseudophakic/phakic/aphakic)	(49/15/2)
Number of hypotensive eye drops	2.6 ± 1.1
Number of acetazolamide tablets	0.7 ± 0.8
**Previous glaucoma treatments (%)**	
Filtering surgery	17 (25.8%)
Selective laser trabeculoplasty	8 (12.1%)
Laser cyclophotocoagulation	3 (4.5%)

**Figure 1:**
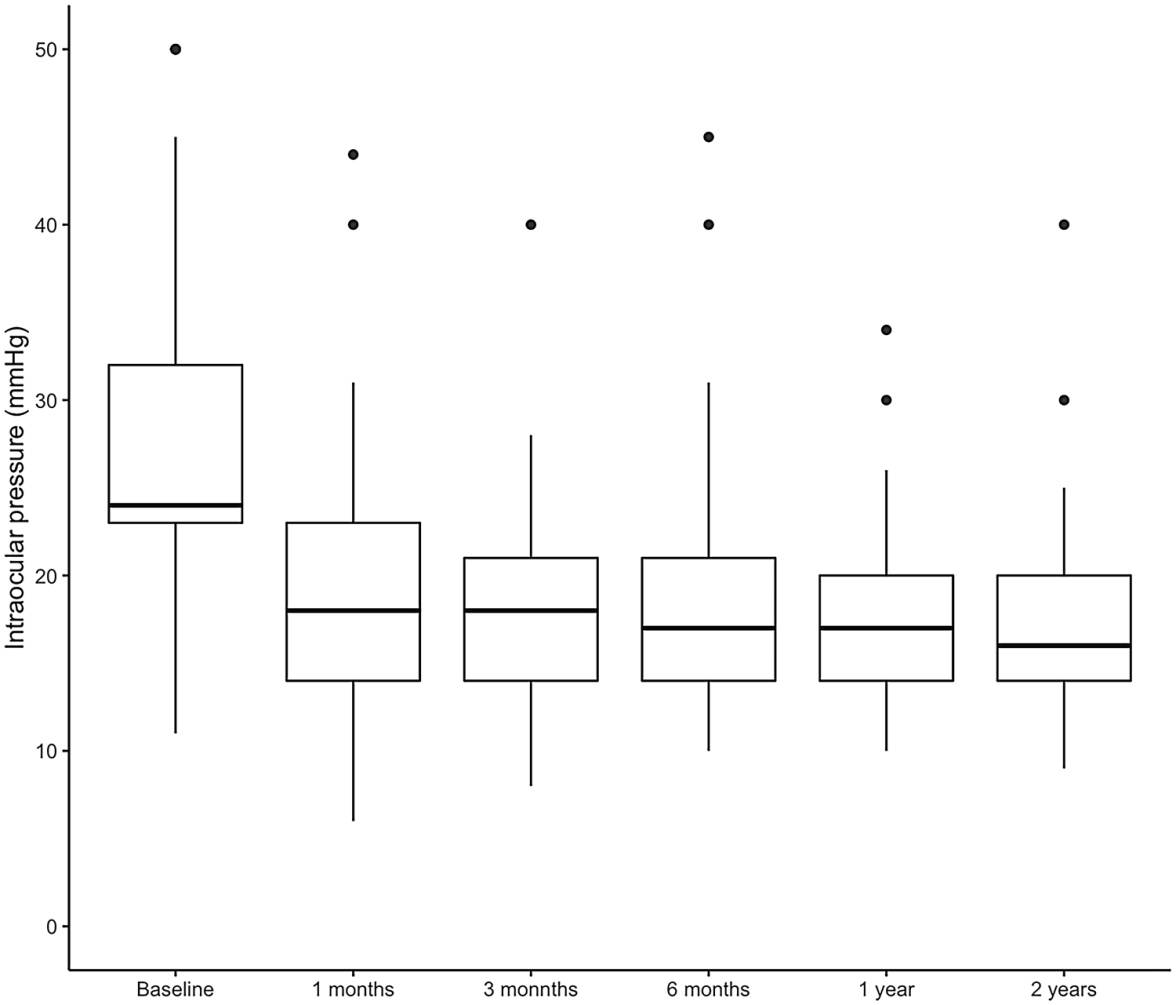
Intraocular pressure values throughout the 2-year follow-up period. The figure was created using R (version 4.0.0) and RStudio (version 1.2.5042) software (https://www.rstudio.com).

Overall, 60 patients competed regularly the 2-year follow-up, while the remaining 6 (9.1%) were lost to follow-up. Mean preoperative IOP was 28.5 ± 9.6 mmHg and significantly decreased to 19.2 ± 7.0 at 1 month, 18.8 ± 7.7 at 3 months, 18.4 ± 6.7 at 6 months, 17.6 ± 5.0 at 1 year and 17.0 ± 5.4 at 2 years (always *p* < 0.001 compared to preoperative values) (Fig. 1). At 2 years, the IOP was significantly lower in patients with primary open angle glaucoma compared to those with other glaucoma types (15.3 ± 3.4 vs 19.2 ± 6.8, *p* = 0.027). The daily number of hypotensive eye drops significantly decreased from 2.6 ± 1.1 at baseline to 1.7 ± 1.2 at the last follow-up (*p* < 0.001); in parallel, also the daily number of 250 mg acetazolamide tablets decreased from 0.7 ± 0.8 to 0.2 ± 0.5 (*p* < 0.001). However, the number of topical and oral hypotensive medications at 2 years did not differ significantly between patients with primary open angle glaucoma compared to those with other glaucoma types (respectively, 1.8 ± 1.2 vs 1.5 ± 1.3, *p* = 0.237; 0.3 ± 0.5 vs 0.2 ± 0.5, *p* = 0.464).

**Figure 2:**
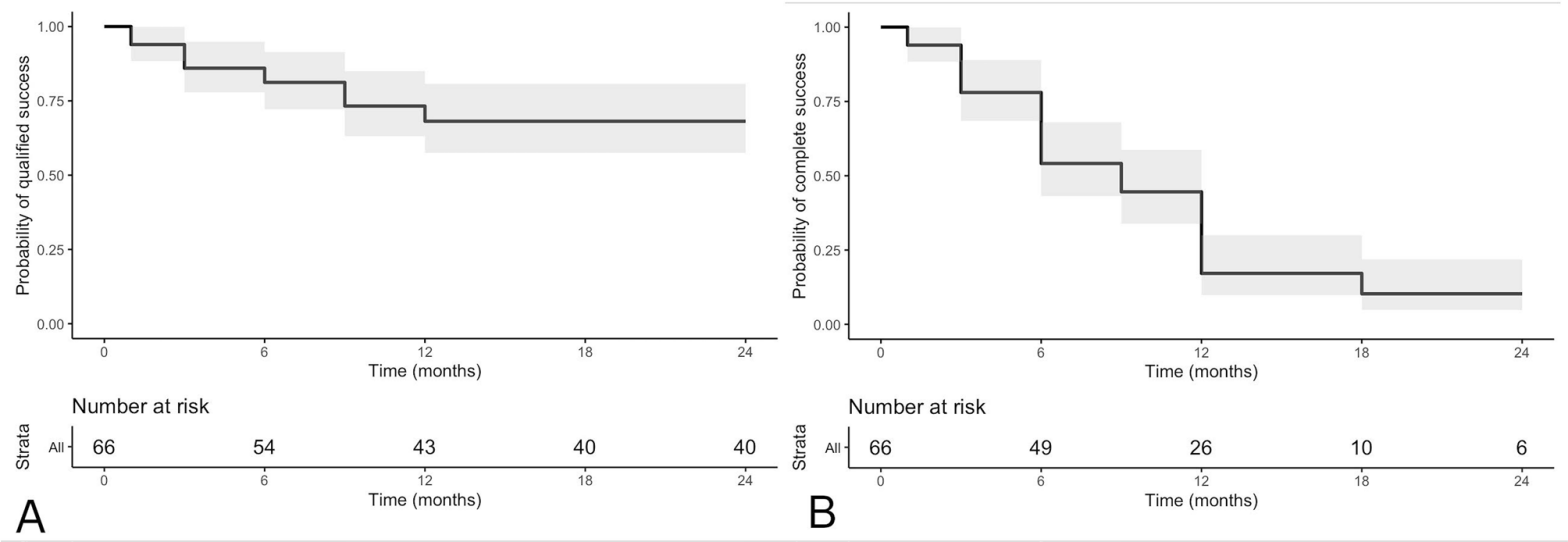
Kaplan–Meier survival curves of qualified success (**A**) and complete success (**B**) after ultrasound cyclo plasty. The figure was created using R (version 4.0.0) and RStudio (version 1.2.5042) software (https://www.rstudio.com).

At 2 years, 68.1% of patients met the definition of qualified success (95% confidence interval 57.5–80.7) (Fig. 2A). Among those who did not reach qualified success, 15 patients required additional surgery (trabeculectomy in 7 eyes, laser cyclophotocoagulation in 6, Ahmed valve implant in 2) while 5 patients had IOP > 21 mmHg but refused further surgery. At 2 years, 10.3% of patients met the definition of complete success (95% confidence interval 4.9–21.9%) (Fig. 2B). Among those who did not reach complete success, 34 patients had IOP < 21 mmHg but the use of hypotensive medications was required.

The factors evaluated for the association with treatment failure are shown in Table 2. The rate of treatment failure was significantly lower in patients with primary open-angle glaucoma (*p* = 0.024) and in those with lower baseline IOP (*p* < 0.001). Conversely, there were no associations between rate of treatment failure and age, gender and previous glaucoma surgery (always *p* > 0.05). After multivariate analysis, an association was maintained between rate of treatment failure and baseline IOP (HR = 1.061, 95% CI 1.017–1.107; *p* = 0.006), while primary open-angle glaucoma was no longer significant (HR = 0.536; 95% CI 0.185–1.556; *p* = 0.251).

**Table 2:** Cox proportional hazard regression analysis of rate of treatment failure.

Predictor	HR	95% CI	*p*
Glaucoma type (POAG vs others)	0.333	0.128 – 0.867	0.024
Baseline IOP	1.074	1.033 – 1.117	< 0.001
Age	0.986	0.957 – 1.016	0.364
Gender	0.510	0.196 – 1.329	0.169
Previous glaucoma surgery	2.2426	0.989 – 5.952	0.053

*HR* hazard ratio, *CI* confidence intervals, *POAG* primary open-angle glaucoma, *IOP* intraocular pressure.

No major intra- or postoperative complications occurred and no patient required an unscheduled visit. Minor complications recorded in the early postoperative period (within 1 month) included conjunctival hyperemia or chemosis (24.2% of the total), superficial punctuate keratitis (12.1%), anterior chamber uveal reaction (9.1%) and transient mild mydriasis (4.5%). Focal areas of scleral thinning (“scleral marks”) were visible throughout the entire follow-up in 4.5% of patients.

## Discussion

In the present study, UCP significantly reduced both IOP values and daily number of hypotensive medications throughout the entire follow-up period. At 2 years, mean IOP reduction from baseline was 40.5%, 68.1% of eyes had controlled IOP and only 15 patients (less than one fourth) underwent additional glaucoma surgery. Furthermore, the procedure was safe, with no major complications recorded in any of the treated eyes. Thus, our results support the use of UCP as a therapeutic option for patients with uncontrolled IOP despite maximum medical therapy or intolerance to glaucoma medications. The results of this study are consistent with several previous studies with shorter follow-up that reported an IOP reduction ranging from 30 to 42%1^11–22^. However, it is difficult to directly compare these studies due to differences in study population, baseline IOP value, postoperative treatment protocol and study design.

The majority of published studies on UCP had a short-to-mid follow-up duration, and long-term data on the efficacy and safety of the procedure are still scarce. To our knowledge, only one recent study reported the 2-year outcomes of UCP performed in 15 eyes with moderate open angle glaucoma^20^. Although surgical success was achieved in 87% of the patients at the last follow-up visits, only 9% met the definition of complete success without the need of any hypotensive medication. In agreement with this finding, we observed a similar rate of complete success (10%) in our sample. Most patients did not reach a complete success due the need for resuming hypotensive medications to better control IOP during the follow-up. The progressive loss of treatment efficacy has been attributed to the gradual re-epithelialization of the ciliary processes with partial recovery of their function^23–25^.

We found a significant association between baseline IOP values and efficacy of UCP, with significantly lower treatment success in patients with higher baseline IOP. This result is not surprising since patients with highly elevated IOP are usually more challenging to treat. Of note, the diagnosis of primary open angle glaucoma was also associated with the success of UCP. However, this association was no longer significant after multivariate adjustment. Thus, it might have resulted from the lower baseline IOP values detected in patients with primary open angle glaucoma.

We did not observe any major intra- or postoperative complications such as severe hypotony, phthisis bulbi, choroidal effusions or hemorrhages or irreversible visual loss. The favorable safety profile of UCP compared to traditional cyclodestructive techniques represents a major advantage of the procedure, allowing the treatment of patients with earlier stages of glaucoma and with good or even full visual acuity. Furthermore, a lower number of postoperative visits compared to traditional glaucoma filtering surgery is needed after UCP. This aspect appears to be particularly crucial in the current scenario of pandemic. In fact, it has been recently proposed that in the COVID-19 era minimally-invasive glaucoma surgeries may be more indicated for controlling IOP compared to incisional surgeries that require a more intense postoperative care, and in some cases also the need for repeated surgery^26^. Of note, in our study no patient required neither unscheduled visits nor repeated surgeries, except for those who experienced treatment failure.

This study suffers from some important limitations that deserve mentioning, including the non-comparative design and the heterogeneous glaucoma population. However, to the best of our knowledge this is the largest study available for UCP technology with a 2-year follow-up.

In conclusion, UCP proved to be an effective and safe non-incisional technique for the treatment of glaucoma. The IOP reduction was maintained relatively stable 2 years after the procedure and no major complications occurred throughout the entire follow-up. The failure rate was found to be higher in patients with higher baseline IOP.

## Methods

This prospective study was conducted in 2 Italian tertiary referral centers (University Eye Clinic in Genoa and Scientific Institute Capitanio Hospital in Milan). Inclusion criteria were age ≥ 18 years, a confirmed diagnosis of glaucoma based on glaucomatous optic nerve damage, either primary or secondary, IOP values ≥ 21 mm Hg despite maximum medical therapy, or had IOP values within the normal range but intolerance to glaucoma medications. Exclusion criteria were pregnancy, diagnosis of normal-tension glaucoma, laser or surgical procedure performed in the last 6 months, ocular infections or uveitis occurred in the last 3 months. Ultrasound cyclo plasty was offered as an alternative to conventional filtering surgery to all patients who met the inclusion and exclusion criteria. The study was approved by the local Institutional Review Boards (Istituto Auxologico Italiano and San Martino University Hospital) and was carried out in accordance with the principles of the Declaration of Helsinki. Written informed consent was obtained from all participants.

All operations were performed by a senior surgeon using the EyeOP1 device (Eye Tech Care, Rillieux-la-Pape, France) under peribulbar anesthesia, according to a technique previously described^26^. Briefly, a coupling cone was placed in contact with the eye, and a suction ring was used to create low level vacuum and stabilize the probe. The diameter of the probe was selected based on preoperative biometric data in order to best adapt to the eye. The cavity between the eye and the cone was filled with balanced salt solution. A ring containing 6 piezoelectric transducers was inserted into the coupling cone. Two generation of probes have been used: the first one allowing to choose between 4 and 6 s of treatment time duration; the second one offered a unique treatment time duration of 8 s for each transducer. The operating frequency was 21 MHz, with an acoustic power of 2–3 W. After surgery, tobramycin and dexamethasone eye drops were administered 4 times a day for 4 weeks while hypotensive medications were interrupted and then prescribed again only if postoperative IOP was found to be ≥ 21 mmHg during a routine follow-up visit.

Postoperative examinations were performed at day 1, 7, month 1, 3, 6, and every 6 months thereafter. Each visit was performed approximately at the same time of the day (from 9 to 11 am) and included best corrected visual acuity (BCVA), IOP measurement with Goldmann applanation tonometry, slit-lamp biomicroscopy and mydriatic fundus examination. As previously described, qualified success was defined as IOP < 21 mmHg regardless of the use of glaucoma medications, while complete success was defined as IOP < 21 mmHg without glaucoma medications^17^. The following cases were classified as failures: eyes with postoperative IOP ≥ 21 mmHg; eyes that required a higher number of glaucoma medications compared to preoperatively; eyes that underwent additional glaucoma surgery.

Statistical analysis was performed using R (version 4.0.0) and RStudio (version 1.2.5042) software. The Wilcoxon test was used to compare the change of IOP and number of hypotensive medications before and after surgery. The probability of treatment success was examined by means of Kaplan–Meier survival curves. Cox proportional hazards regression analysis was used to determine the association between baseline characteristics and the probability of treatment success. Patients who were lost to follow-up were treated as censored in the Cox regression analysis. Variables evaluated were age, gender, glaucoma type, baseline IOP and previous glaucoma surgery. A *p* value of less than 0.05 was considered statistically significant.
